# An improved schlieren method for measurement and automatic reconstruction of the far-field focal spot

**DOI:** 10.1371/journal.pone.0171415

**Published:** 2017-02-16

**Authors:** Zhengzhou Wang, Bingliang Hu, Qinye Yin

**Affiliations:** 1 School of Electronic & Information Engineering, Xi'an Jiaotong University, Xi’an, China; 2 University of Chinese Academy of Sciences, Beijing, China; 3 Xi’an Institute of Optics and Precision Mechanics Chinese Academy of Science, Xi’an, China; Universita degli Studi di Milano-Bicocca, ITALY

## Abstract

The schlieren method of measuring far-field focal spots offers many advantages at the Shenguang III laser facility such as low cost and automatic laser-path collimation. However, current methods of far-field focal spot measurement often suffer from low precision and efficiency when the final focal spot is merged manually, thereby reducing the accuracy of reconstruction. In this paper, we introduce an improved schlieren method to construct the high dynamic-range image of far-field focal spots and improve the reconstruction accuracy and efficiency. First, a detection method based on weak light beam sampling and magnification imaging was designed; images of the main and side lobes of the focused laser irradiance in the far field were obtained using two scientific CCD cameras. Second, using a self-correlation template matching algorithm, a circle the same size as the schlieren ball was dug from the main lobe cutting image and used to change the relative region of the main lobe cutting image within a 100×100 pixel region. The position that had the largest correlation coefficient between the side lobe cutting image and the main lobe cutting image when a circle was dug was identified as the best matching point. Finally, the least squares method was used to fit the center of the side lobe schlieren small ball, and the error was less than 1 pixel. The experimental results show that this method enables the accurate, high-dynamic-range measurement of a far-field focal spot and automatic image reconstruction. Because the best matching point is obtained through image processing rather than traditional reconstruction methods based on manual splicing, this method is less sensitive to the efficiency of focal-spot reconstruction and thus offers better experimental precision.

## Introduction

The output parameters of a large laser facility, including the distribution in the time domain, the distribution in the spatial domain, the distribution in the frequency domain, the temporal distribution of the wave parameters, and the distribution in the far field, which differ for different experimental requirements, are closely related to the input energy. As a result, the distribution in the far field prevents the entire focal spot from being measured during a shooting experiment when the laser energy is at approximately the 10 kJ level [1]. The distribution in the far field plays a key role in the measurement of the far-field focal spot. There are two purposes of measuring the precise focal spot in a laser facility [2]. The first purpose is to determine the influence of the optical components on the far-field focal spot under different input power conditions [3]. The second purpose is to provide a basis for improving the quality of the optical components once the spatial distribution of the focal spot has been obtained [4]. However, the size and shape of the focal spot can only be improved when the position of the optical element is adjusted or when the structure of the laser path is modified by the designer.

An integrated diagnostic system is a multifunctional laser parameter system whose main functions are running the device during emission; completing the precision diagnosis of laser beam energy, near-field, far-field, pulse waveform and spectrum parameters; and obtaining the device operating parameters comprehensively and accurately. The most important task of the integrated diagnosis system is to construct the high-dynamic-range image of the far-field focal spot [5]. The high-dynamic-range measurement of the focal-spot distribution always poses a difficult problem in diagnosing the parameters of a large laser facility and represents the key technology that must be addressed for high-power physical experiments.

At present, many scholars have made many significant contributions to research on methods of measuring the distribution of a far-field focal spot; the main methods include the array camera method, the diffraction grating method and the schlieren method. In 2012, Reyad Mehfuz developed the array camera method to test a far-field focal spot based on images exposed for different times to reconstruct a high-dynamic-range image [6]. However, because a physical experiment at a high-power laser facility is a high-throughput targeting experiment, it is difficult to achieve multiple exposures in such a targeting experiment with the high precision required to measure the laser intensity. HE Yuanxing et al proposed a far-field focal spot reconstruction method based on a diffraction grating [7]. The far-field laser spot is divided into two spots when such a grating is used, and the side lobe is submerged in noise and cannot be measured; thus, the main lobe must be blocked to detect the side lobe spot. Because the side lobe and main lobe are captured by the same CCD and because the main lobe is sheltered by a small schlieren ball, there is a large effect on the side lobe data that makes it very difficult to separate the side lobe data from the main lobe data. Because the dynamic range of the far-field focal spot measured by an integrated diagnosis system is up to 1000:1, it is very difficult for a single detector to meet the requirements of such a high dynamic range. Because the energy during emission reaches up to 10 kJ, if there is no large attenuation in the laser paths, the CCD used for detection will be completely saturated. If the attenuation is too large, although the far-field focal spot can be tested, the signal bottom of the focal spot will be submerged in noise. In the last year, a considerable amount of work has been done regarding schlieren. The application of different schlieren methods has resulted in great progress in many fields. For example, rainbow schlieren deflectometry has been employed to record the projection data of the temperature gradient field in test cavities [8]. Background-oriented schlieren (BOS) uses correlation techniques on a background dot pattern to characterize thermal flows with good spatial and temporal resolution [9]. Laser schlieren deflectometry (LSD) has been successfully employed as a temperature measurement method to reveal heat convection [10]. A non-scanning 3D-CT (Computer Tomography) technique using a multi-directional quantitative schlieren system with a flash light source was proposed for the instantaneous density distribution of unsteady premixed flames [11]. Despite the latest development mentioned above, one of the most classical applications of the schlieren method is measuring the far-field distribution of a high-power laser [5]; in this method, the main lobe and side lobe are measured on two separate laser paths, the side lobe is sheltered using a small schlieren ball, the intensity of the main lobe is magnified a given number of times, and the main lobe and side lobe data are merged to obtain a high-dynamic-range far-field focal spot. The schlieren method was proposed for far-field focal spot measurement at the National Ignition Facility in America [1]. Because of the great differences in light energy density between the main lobe and side lobe of the focal spot, there is considerable drift when the laser beam is transmitted long distances; thus, the image quality of the main lobe and side lobe images is poor, meaning that the reconstructed image cannot be stitched accurately. The schlieren method has also been adopted for far-field focal spot measurement at Shenguang III in China. However, the conventional method used to reconstruct the image of the far-field focal spot is stitched manually, even though the texture is very clear and the quality is very good [12], resulting in a very low experimental efficiency.

Many investigators have studied the schlieren reconstruction algorithm. Cheng et al. introduced a far-field measurement principle using the schlieren method [13], but their principle lacks a detailed reconstruction step or mathematical model and cannot obtain the reconstructed focal spot automatically.

As a result, we conclude that, to achieve a robust measurement of the far-field focal spot under conditions of high input energy, low experimental efficiency and low reconstruction accuracy should be avoided, and the manual reconstruction steps that have been widely used in previous experiments are no longer suitable. In this paper, an improved algorithm for far-field focal spot measurement and automatic reconstruction that achieves good performance in terms of accuracy and efficiency is proposed. The detailed steps of the proposed method are summarized as follows. First, a weak light beam sampling and magnification imaging detection method is designed, and the main lobe and side lobe beam spots are captured by two scientific CCD cameras. Second, using a self-correlation template matching algorithm [14], the position for which the largest correlation coefficient between the side lobe image and the main lobe image with a circle dug is identified as the best matching point, enabling the automatic reconstruction of the main lobe and side lobe into a mosaic image. Then, the weighted average method is used to fuse the splicing edge. Finally, the least squares method is used to fit the center of the side lobe schlieren small ball, with an error of less than 1 pixel. The method applied to obtain the reconstructed image in the splice region texture achieves excellent agreement, with a registration error of less than 1 pixel; it can not only obtain the complete far-field image but also meet the efficiency requirements for targeting experiments. It will be important and significant for the ability of integrated diagnostic systems to assimilate laser parameters accurately and completely.

## Methods

### The principle of the measurement of the far field

**Fig 1:**
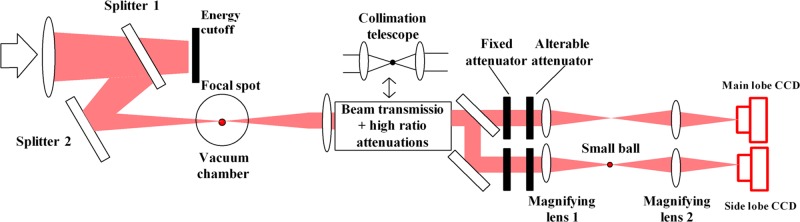
Schematic diagram of the laser beam focal plane measurement using the schlieren method.

To evaluate the focusing performance of different shooting lenses in a 10 kJ-level laser facility [15], a weak laser is sampled first. The laser is transmitted together in the front beam and divided into two beams after high-ratio attenuations. The two beams are then captured by two CCD cameras. The principle of the optical system is shown in Fig 1. The beam transmitted in the front of the small vacuum target chamber is the sampling optical path, and the beam transmitted in the back of the target chamber is the diagnostic optical path. The converging laser beam is transmitted from the wedge shooting lens, reflected by two wedge mirrors, and transmitted into the small vacuum target chamber. The small target chamber center is regarded as a benchmark, and the position of the shooting lens can be moved forward or backward according to the different focal lengths to ensure that the metering spot is located in the small target chamber center, thus completing the sampling of the focal spot. Most of the energy across the first sampling mirror transmission is absorbed by the energy cutoff, and the reflected weak light is transmitted into the vacuum small target chamber; thus, a huge vacuum system is avoided, ensuring that assessing the focal spot will be convenient.

The beam emitted from the center of the small target chamber is collimated to a small-diameter parallel laser by the transmission system. The energy of the laser will be attenuated by a factor of approximately one million after the beam crosses the high-rate attenuator. The beam is divided into the main lobe optical path and the side lobe optical path by a beam splitter. In the side lobe optical path, the parallel beam is first converged by magnifying lens 1, and the focus is magnified by magnifying lens 2. The small schlieren ball is placed at the focal point of magnifying lens 1 to shelter the focal spot center of the main lobe, and thus, the image captured by the side lobe CCD contains most of the laser spot information surrounding the small schlieren ball. In the main lobe optical path, the beam is attenuated appropriately, and the image captured by the main lobe CCD contains the laser spot information without blocking. To ensure consistency of the main lobe and side lobe in terms of optical aberrations, the main lobe optical path is similar to the side lobe optical path. The parameters of the two optical paths must be consistent in terms of the number and thickness of the attenuation component; the only difference is in the attenuation rate of each component. Therefore, the different far-field focal spots can be measured only when the appropriate magnifying lens group is replaced according to the different input energy, and the requirements of the facility can be met under different experimental conditions.

The automatic collimation system can be divided into the collimation system of the small target chamber and the focal spot optical path. The purpose of the front collimation system is to converge the sampling beam to the simulated target point. The first purpose of the collimation system located in the front small target chamber is to capture the accurate far-field laser spot of the simulated target point by the main lobe and side lobe CCDs and obtain the correct distribution of the far-field focal spot. The second purpose of the collimation system is to calibrate the parameters of the schlieren method, including the magnification ratio *k* of the main lobe intensity, the magnification ratio *b* of the laser spot image, and the relative position between the laser spot of the main lobe and the laser spot of the side lobe [5].

### The mathematical model of the schlieren method

The mathematical model of the schlieren method for measuring the far-field focal spot was proposed in the literature [5]. The reconstructed image can be expressed as the following function:

$$h\left(x,y\right)=\{\begin{array}{ll} Kh_{1}\left(bx,by\right) & \left(x,y\right)\in A\\ h_{2}\left(x,y\right) & \left(x,y\right)\in B\\ d_{1}Kh_{1}\left(bx,by\right)+d_{2}h_{2}\left(x,y\right) & \left(x,y\right)\in A\cap B=C\neq \emptyset \end{array}$$ (1)

where the main lobe image function is *h*_1_(*x*, *y*), the domain is (*x*, *y*) ∈ *A*, the side lobe image function is *h*_2_(*x*, *y*), and the domain is (*x*, *y*) ∈ *B*. With the side lobe image as a reference, the main lobe intensity lobe is magnified *K* times (*K*>1), the main lobe is magnified *b* times (*b*>1), and the main lobe image is filled into the side lobe schlieren ball area. A certain proportion of the overlap edge is occupied by the main lobe and side lobe image.

**Fig 2:**
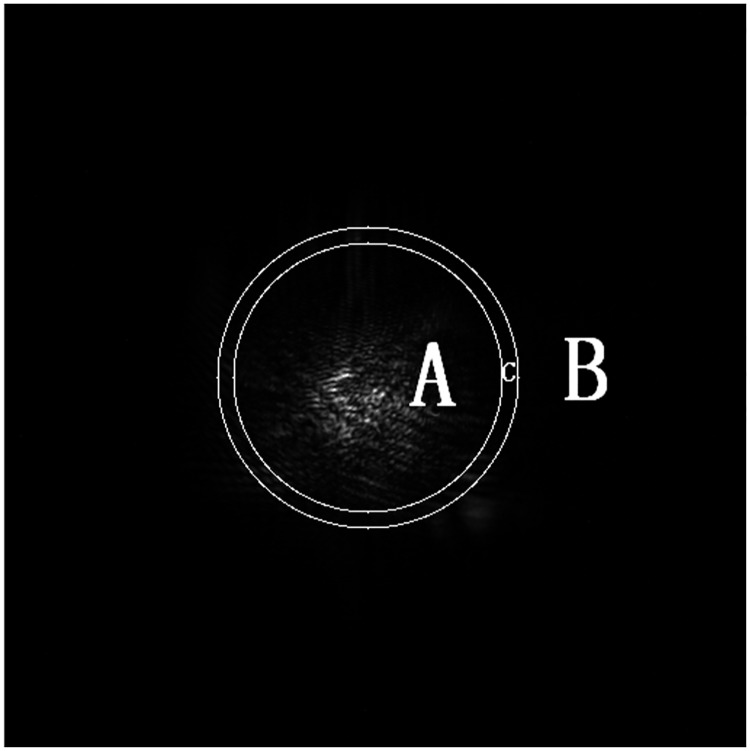
The reconstructed region using the schlieren method. Region C is the region between the smaller and larger circles and is a transitional region with a width of 10 pixels.

The data of region A are replaced by the main lobe spot and are magnified *K* times, the data of region B are replaced by the side lobe spot, and the data of region C are overlapped by the main lobe spot and the side lobe spot. Region C is the transition zone and is shown in Fig 2; region A is the smaller inner ring region, and region B is the larger outer ring region. Region C is a transitional region with a width of 10 pixels and is used to fuse the splicing boundary of the merged image by the weighted average method. The radius of the smaller circle is the radius of the small schlieren ball minus 5 pixels, and the radius of the larger circle is the radius of the small schlieren ball plus 5 pixels. *d*_*1*_ and *d*_*2*_ represent the relative proportions of the image belonging to the overlapping main lobe and side lobe regions, respectively, where *d*_*1*_ + *d*_*2*_ = 1, 0< *d*_*1*_<1 and 0< *d*_*2*_<1.

To calibrate the intensity magnification ratio *K* of the main lobe and the magnification ratio *b* of the laser spot, we designed a new collimation telescope component (i.e., a telescope and a small ball, as shown in Fig 1) in the integrated diagnosis system. The steps to calibrate *K* and *b* are as follows.

1. The small schlieren ball is moved out of the laser beam, and the collimation telescope is moved into the laser beam. The images of the small telescopic ball are captured by the side lobe and main lobe CCDs, and the center coordinates of the small telescopic ball are (*A*_1*X*_, *A*_1*Y*_) and (*B*_1*X*_, *B*_1*Y*_).
2. The small schlieren ball is moved a tiny distance. The new center coordinates of the telescopic ball image reflected in the side lobe CCD and main lobe CCD are (*A*_2*X*_, *A*_2*Y*_) and (*B*_2*X*_, *B*_2*Y*_), respectively, and the magnification ratio of the main lobe CCD relative to the side lobe CCD is *b*, which is the ratio of the distance between (*B*_1*X*_, *B*_1*Y*_) and (*B*_2*X*_, *B*_2*Y*_) to the distance between (*A*_1*X*_, *A*_1*Y*_) and (*A*_2*X*_, *A*_2*Y*_).
3. To calibrate the intensity magnification ratio *K*, a standard laser source is inserted into the position of the small telescopic ball and captures the main lobe and side lobe images more than 20 times. The average gray level of the side lobe image divided by that of the main lobe image is given by *K*.

### The schlieren automatic reconstruction algorithm

In previous experiments, the common method used to obtain the reconstructed image has been manual splicing [13]. The corresponding coordinates of the schlieren ball center in the main lobe image are the most important parameters and are found using the manual method through repeated experimentation. To achieve automatic reconstruction of the far-field focal spot, a new method based on a self-correlation template matching algorithm is proposed in this paper because the following characteristics between the main lobe and side lobe images are observed: 1) a circular schlieren ball can be found in the side lobe image center, and 2) if a circle with the same size as the schlieren ball is dug from the main lobe image, the main lobe and side lobe images have very similar characteristics. If the schlieren ball dug from the main lobe image is moved within a certain range, when the correlation coefficient between the main lobe and side lobe images is maximal—that is, the two images present maximal similarity—the corresponding coordinates of the schlieren ball center in the main lobe image is finally found. Only when the schlieren ball region of the side lobe image is replaced by the data dug from the main lobe image and magnified *K* times will it be possible to obtain the best reconstructed image.

In the search for the optimal splicing position, a self-correlation template matching algorithm based on self-correlation matching theory is used [14]; the correlation coefficient of the two images is calculated as shown in formula (2):

$$r\left(x,y\right)=\frac{\sum_{s=0}^{L}\sum_{t=0}^{J}w\left(s,t\right)f\left(x+s,y+t\right)}{{\left[\sum_{s=0}^{L}\sum_{t=0}^{J}w^{2}\left(s,t\right)\cdot \sum_{s=0}^{L}\sum_{t=0}^{J}f^{2}\left(x+s,y+t\right)\right]}^{1/2}}$$ (2)

where *f (x*, *y)* represents the image of size m × n, *w (x*, *y)* represents the sub-images of size *J× L*, and x = 0,1,2,…, n-*L*, y = 0,1,2,…, m-*J*. In the automatic reconstruction algorithm provided in this paper, *f (x*, *y)* represents the side lobe image, *w (x*, *y)* represents the main lobe image from which a circle with the same size as the schlieren ball is dug, and the size of the two images is 300 × 300.

### Experiment

The schlieren automatic reconstruction algorithm contains several important steps: preprocessing, obtaining the schlieren ball center by circle fitting, searching for the best matching point of the schlieren ball center in the main lobe image, and merging the main lobe and side lobe images. Among these, the critical step is to search for the best matching point of the schlieren ball center in the main lobe image (S1 File). The automatic reconstruction algorithm for this step is described briefly as follows.

Algorithm 1 schlieren automatic reconstruction algorithm

Input: *orgpb* image, *orgzb* image

1 Obtain the center of gravity *orgzb* and radius of small schlieren ball in *orgpb*.

2 Obtain the *cutpb* image

3 for m = -50: 50–1

 for l = -50: 50–1

  Obtain a *cutzb* image

  Dig a circle region from *cutzb* image

 end for

end for

4 Obtain the best matching position

5 Merge the image with the best matching *cutzb* image and *cutpb* image

Output: The reconstructed image

where orgz*b* is the original main lobe image of size 512×512, org*pb* is the original side lobe image of size 512×512, *cutzb* is the cutting main lobe image of size 300×300, and *cutpb* is the cutting side lobe image of size 300×300.

#### Preprocessing

**Fig 3:**
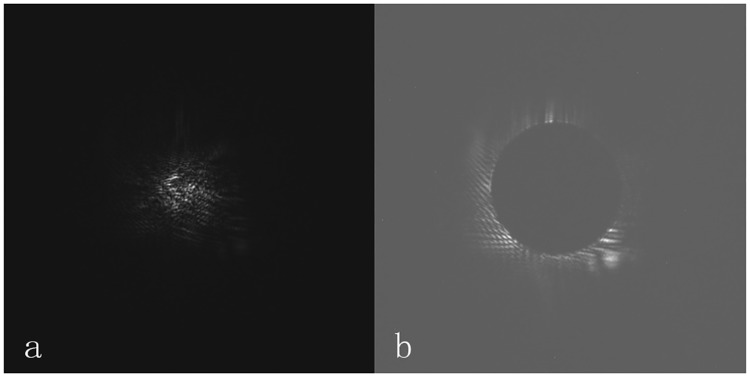
Images clipped for the first time. (a) The *orgzb* of the main lobe. (b) The *orgpb* of the side lobe.

Before the image is reconstructed, the background must be subtracted from the original main lobe and side lobe images. Second, the image captured by the CCD must be clipped because two 14-bit scientific CCDs with a resolution of 1024 × 1024 are used in the ICF experiment, and the best match point is searched by an automatic algorithm. Another reason to clip the image is that the size of the image captured by the CCD is larger than the size of the laser spot such that the amount of computation will be reduced during the reconstruction process when the images are clipped. The size of the clipped image is 512×512. The clipped images of the main and side lobes are recorded as *orgzb* and *orgpb*, respectively, and the laser spot is located as close to the center of the image as possible, as illustrated in Fig 3.

#### Calculating the center of the schlieren ball in the side lobe image

**Fig 4:**
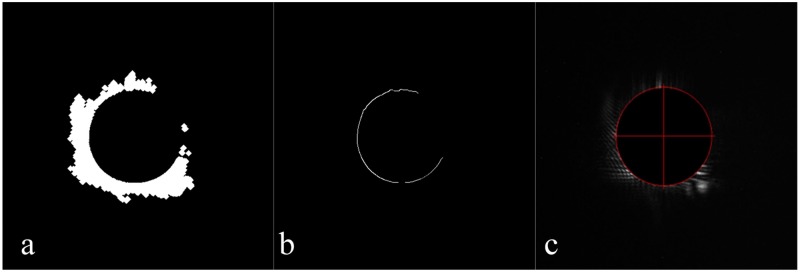
Calculation of the center of the schlieren ball in the side lobe image. (a) The clipped image of the side lobe processed by the morphological operation. (b) The edge of the small ball detected using the Sobel operator. (c) The final center and radius fitted using the circle fitting method.

To calculate the center of the schlieren ball, the edge of the schlieren ball must first be obtained using the Sobel operator [15], and then, the least squares method can be used to fit a circle with high fitting precision. According to the literature [16], the circle fitting formula is

$$\left\{ \begin{array}{l} x_{0}=\frac{\left(\bar{x^{2}}\bar{x}+\bar{x}\bar{y^{2}}-\bar{x^{3}}-\bar{xy^{2}}\right)((\bar{y}{)}^{2}-\bar{y^{2}})-(\bar{x^{2}}\bar{y}+\bar{y}\bar{y^{2}}-\bar{x^{2}y}-\bar{y^{3}})(\bar{x}\;\bar{y}\;-\bar{xy})}{2((\bar{x}{)}^{2}-\bar{x^{2}})((\bar{y}{)}^{2}-\bar{y^{2}})-2(\bar{x}\;\bar{y}-\bar{xy}{)}^{2}}\\ y_{0}=\frac{\left(\bar{x^{2}}\bar{y}+\bar{y}\bar{y^{2}}-\bar{y^{3}}-\bar{x^{2}y}\right)((\bar{x}{)}^{2}-\bar{x^{2}})-(\bar{x^{2}}\bar{x}+\bar{x}\bar{y^{2}}-\bar{xy^{2}}-\bar{x^{3}})(\bar{x}\;\bar{y}\;-\bar{xy})}{2((\bar{x}{)}^{2}-\bar{x^{2}})((\bar{y}{)}^{2}-\bar{y^{2}})-2(\bar{x}\;\bar{y}-\;\bar{xy}{)}^{2}}\\ r=\sqrt{x_{0}^{2}-2\bar{x}x_{0}+y_{0}^{2}-2\bar{y}y_{0}+\bar{x^{2}}+\bar{y^{2}}} \end{array}\right.$$ (3)

$$\bar{x^{m}y^{n}}=\frac{1}{N}\sum_{i\in E}x_{i}^{m}y_{i}^{n}$$ (4)

where N is the total number of boundary points, (*x*_*i*_, *y*_*i*_) represents the boundary coordinates of the side lobe image, and r is the radius. (*x*_0_, *y*_0_) is the center of the schlieren ball; the center and radius of the schlieren ball in the side lobe image obtained from the circle fitting formula are (*Opx*, *Opy*) and *okr*, respectively, and the side lobe data within the radius of the schlieren ball are set to zero. The code for calculating the center and radius using the circle fitting method is described in the supplemental information file (S1 File). The steps used to calculate the schlieren ball center are as follows:

1. The clipping side lobe image is processed by the mathematical morphology [5], the result of which is shown in Fig 4(a).
2. The edge of the small ball is detected using the Sobel operator [15]; the circle contour is shown in Fig 4(b).
3. The circle contour is used to fit the circle using formula (3), and the result is shown in Fig 4(c).

#### The search for the best matching point of the schlieren ball center in the main lobe image

**Fig 5:**
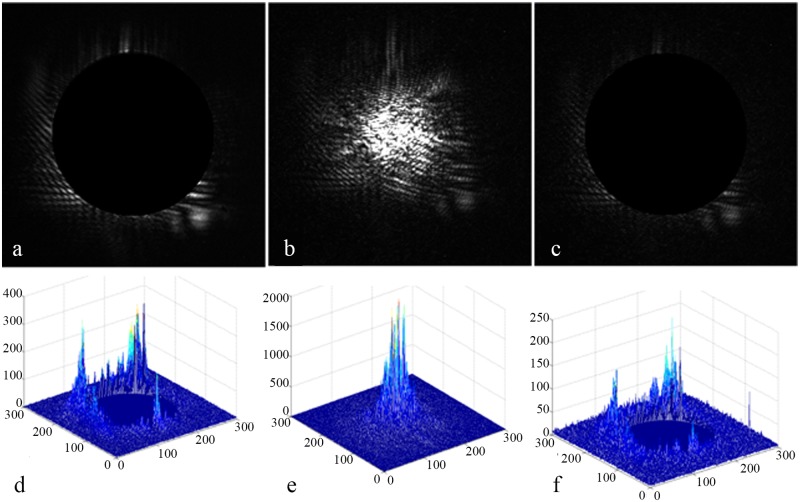
The best matching point for the center of the schlieren ball in the main lobe image. (a) The *cutpb* image. (b) The *cutzb* image. (c) The best matching with the schlieren ball dug from the main lobe image. (d) Three-dimensional *cutpb* image. (e) Three-dimensional *cutzb* image. (f) Three-dimensional image of the best matching with the schlieren ball dug from the main lobe image.

To greatly reduce the time consumption when finding the best matching point of the schlieren ball center in the main lobe image, the main lobe and side lobe image must be clipped a second time, and the two images, with a size of 300×300, are denoted as *cutzb* and *cutpb* (Fig 5).

The steps used to identify the best matching points are as follows:

1. Obtain the center of gravity (*Ozx*, *Ozy*) of *orgzb*. Obtain the center (*Opx*, *Opy*) and radius *okr* of the small schlieren ball in *orgpb* using the circle fitting method.
2. Obtain the *cutpb* image of size 300×300, where the *cutpb* image is cut from the *orgpb* image, with the center located at (*Opx*, *Opy*). The center of *cutpb* is (*Cpx*,*Cpy*), which is equal to (150, 150), as shown in Fig 5(a).
3. Obtain a matrix *Pcir* with size of 300×300, which marks the region of the small schlieren ball of *cutpb*. *Pcir* (i,j) is set to 0 when (i,j) is located within the region of the small ball of *cutpb*; otherwise, *Pcir*(i,j) is set to 1.
4. Obtain the *cutzb* image with size of 300×300. The *cutzb* image is cut from the *orgzb* image, with the center located at (*Ozx+l*, *Ozy+m*), where *l* and *m* are the relative coordinates referring to the center of the rectangle region with size of 100×100. The *l* value range is −50 ≤ *l* < 50, and the *m* value range is −50 ≤ *m* < 50.

**Fig 6:**
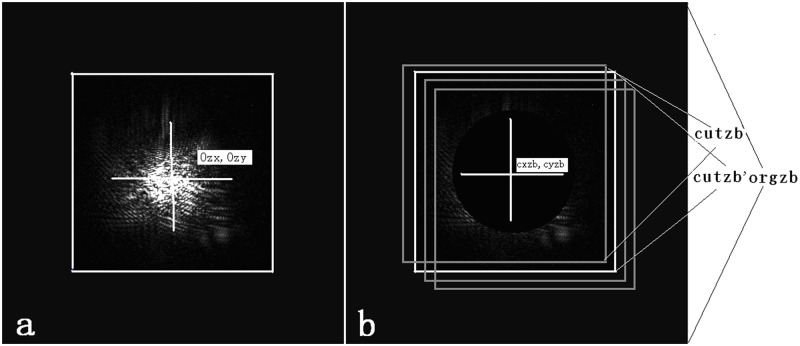
Calculation of the correlation between *cutzb* and *cutpb*. (a) The clipped region of the second clipped main lobe image in *cutzb*. (b) Identification of the best matching position, for which the correlation coefficient between the side lobe image and the main lobe image with a circle dug is maximal.

The white rectangle shown in Fig 6(a) and 6(b) is the real region of *cutzb*, with a size of 300 × 300. A circle region is dug from the new *cutzb* image, and *cutzb* (i,j) is set to 0 when *Pcir* (i,j) is equal to 0.

When *l* and *m* are changed from -50 to 49, the correlation coefficient between *cutzb* and *cutpb* is calculated using formula (2). When the correlation coefficient is maximized, the corresponding coordinates of the best matching position in the *orgzb* image are (*Ozx+l*, *Ozy+m*), and the maximal correlation coefficient is 0.8446. Thus, the main lobe cutting image surrounded by the white rectangle shown in Fig 6(b) is the best matching image of *cutzb* according to *m*,*l*, which is the final cutting image with size 300×300 and is denoted by *cutzb'*. The coordinates of the small schlieren ball in *cutzb’* are (*cxzb*,*cyzb*).

**Fig 7:**
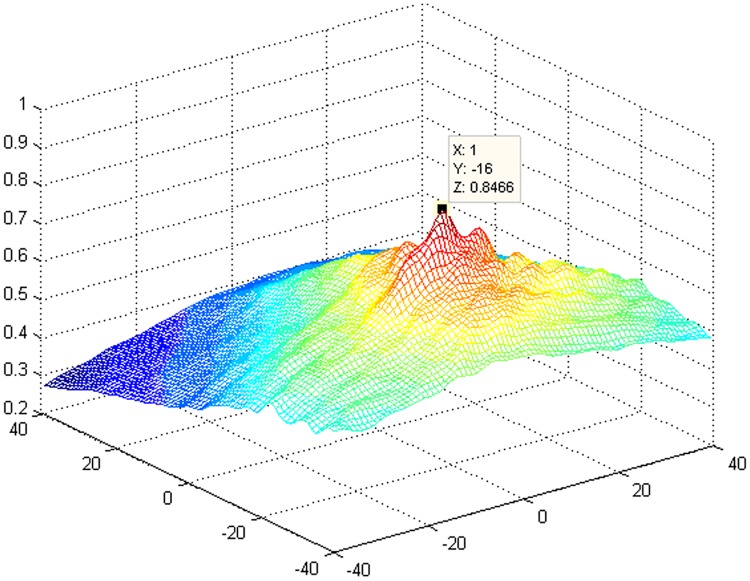
Three-dimensional diagram of the correlation coefficients between *cutpb* and all *cutzb* images.

Between *cutpb* and all *cutzb* images for which (*Ozx+*m, *Ozy+m*) is regarded as the center of the 100×100 region, the three-dimensional diagram of the correlation coefficient is shown in Fig 7. The figure shows that the positions corresponding to the maximum correlation coefficients *m* and *l* are -16 and 1, respectively. The code of the schlieren automatic reconstruction algorithm is presented in the supplemental information file (S1 File).

#### Image merging

The steps of the image merging process are as follows:

1. Fill the small ball region of *cutpb*. The data of *cutpb* located within the region of the small ball are replaced by the data of *cutzb’* when *Pcir*(i,j) is equal to 0 and are magnified *K* times. The notation is *cutpb’*.
2. The region of *cutpb* in *orgpb* is replaced by the final data of *cutpb’* when *Pcir*(i,j) is equal to 0; thus, the new *orgpb* is the final merged image. The notation is *Imerge*.
3. Fuse the splicing boundary [17] by the weighted average method.

**Fig 8:**
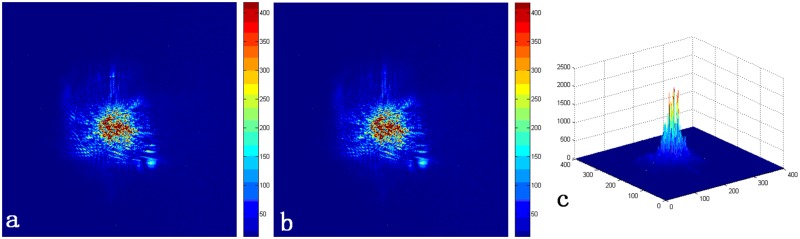
The merged image with a size of 512×512. (a) The directly merged image under the stretching effect. (b) The final merged image under the stretching effect. (c) The final three-dimensional reconstructed image.

Because the edge of the small schlieren image is irregular, the real merging radius is increased by 5 pixels with respect to *okr* to eliminate the splicing mark in the merged image. The direct merging result is shown in Fig 8(a), the merging result of boundary fusion is shown in Fig 8(b) [18]. The final three-dimensional reconstructed image is shown in Fig 8(c).

## Results and discussion

### Analysis of the texture characteristics of the splicing region

**Fig 9:**
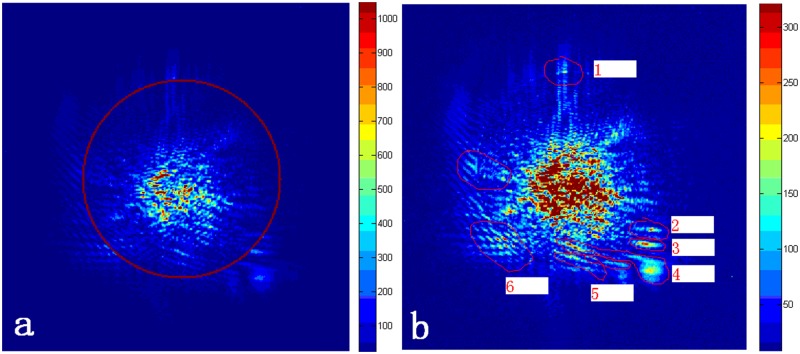
Analysis of the texture characteristics of the splicing region. (a) The position of the splicing ring. (b) The mosaic feature area.

To verify the merging performance of automatic reconstruction, the texture features of the splicing edge are first analyzed. Because the main lobe image is spliced in the side lobe image when obtaining the reconstructed image, the splicing ring is shown in Fig 9(a). The merged image of the inner ring is filled with main lobe image data, and the merged image of the outer ring is filled with side lobe image data. As shown in Fig 9(b), the texture of the six selected regions is consistent. Among them, the stitching error of region 1 in the left and right directions is less than one pixel, the stitching error of regions 2 and 3 in the up and down directions is less than one pixel, and the texture of regions 4–6 is completely consistent; thus, the stitching precision of the reconstruction meets the experimental requirements.

To verify the splicing accuracy of the reconstructed image, the correlation coefficient between the reconstructed image and the main lobe image is 0.997 when the magnification ratio of the main lobe intensity relative to the side lobe intensity *K* = 1; thus, the reconstruction algorithm can be considered to be true and reliable. The means of the reconstructed image and the main lobe image are 34.621 and 35.735, respectively, and the variances are 65.810 and 65.099, respectively, indicating great similarity between the reconstructed image and the main lobe image.

### Improvement of the precision of the center of the small schlieren ball

To improve the precision of the center of the small schlieren ball, formula (3) is used to calculate the schlieren ball center by the fitting method, and the real center and radius are obtained by manual calibration. Experimental data from 5 groups were used to analyze the precision of the center of the small schlieren ball between the circle fitting and the gravity method. The error comparison between the real center and the center of the two algorithms is shown in Table 1. As shown in the table, the error between the center obtained using the circle fitting method and the real center is (0.3, 0.36) pixels, and the error between the center of gravity method and the real center is (1.7, 1.5) pixels, demonstrating that the circle fitting algorithm is better than the gravity method and that the side lobe image center provides greater accuracy. Thus, the authenticity of the reconstructed image can be guaranteed. However, the center and radius of the small schlieren ball can be calculated using the fitting method as long as the circular boundary points are circulated only once. The time complexity of the algorithm is *O(n)*, the calculating time is 0.8 s, and the time consumption is reduced over the whole reconstruction process.

**Table 1:** Error comparison between the fitting method and the gravity method for calculating the center of the schlieren small ball (unit: pixel).

No.	The true center calibrated manually	Center of the fitting method	Center of the gravity method	Center error of the fitting method	Center error of the gravity method
1	X:250.50 Y:254.50	X:251.16 Y:254.79	X:251.52 Y:255.11	X:0.66 Y:0.29	X:1.02 Y:0.61
2	X:251 Y:257.50	X:251.36 Y:257.89	X:252.36 Y:259.29	X:0.36 Y:0.39	X:1.36 Y:1.79
3	X:254 Y:253	X:254.51 Y:253	X:256.10 Y:254.81	X:0.51 Y:0	X:2.10 Y:1.81
4	X:250.50 Y:254.50	X:250.39 Y:255.07	X:252.30 Y:256.85	X: -0.11Y:0.57	X:1.8 Y:2.35
5	X: 248 Y:255	X:248.11 Y:255.54	X:250.07 Y:256.15	X:0.11 Y:0.54	X:2.07 Y:1.15

There is a certain deficiency in obtaining the center of the schlieren ball: the small schlieren ball is placed at a fixed position to block the main lobe spot center when capturing the side lobe image, but the schlieren ball cannot block the side lobe spot center in the actual experiment. Therefore, an automatic alignment system must be designed to improve the positioning accuracy of the schlieren ball such that a perfectly sheltered side lobe image will ultimately be obtained.

### Analysis of the calculations of the corresponding coordinates of the schlieren ball center in the main lobe image

To ensure the precision of the schlieren reconstruction, the most important factor is to accurately calculate the corresponding coordinates of the center of the schlieren ball in the main lobe image. The general methods for calculating the corresponding coordinates of the schlieren ball center in the main lobe image are the center of gravity method [13], the geometrical center method [13], the calibration method [4], and the self-correlation matching method [14], among others. The reconstruction errors of these 4 algorithms are shown in Table 2. The theoretical values of the corresponding coordinates in the shooting experimental data are (249.38, 272.68). The errors between corresponding coordinates obtained using 4 algorithms and the theoretical values are analyzed as follows: The errors of the center of gravity method and the geometrical center method are maximal, namely, greater than 10 pixels in the vertical direction. The error of the calibration method is smaller, namely, more than one pixel. Finally, the error of the autocorrelation matching method is minimal, namely, less than 1 pixel.

**Table 2:** Reconstruction error of the 4 algorithms.

	Corresponding coordinate center	Errors between corresponding coordinates and theoretical values	Correlation coefficients of reconstructed and main lobe images
Center of gravity method	X:251.6 Y:259.6	X:2.22 Y: -13.08	0.629
Geometric center method	X:248.6 Y:261.3	X: -0.78 Y:-11.38	0.615
Calibration method	X:251 Y:272	X:1.62 Y: -0.68	0.806
Self-correlation matching method	X:249 Y:273	X:-0.38 Y:0.32	0.994

By calculating the correlation coefficient between the reconstructed and main lobe images, the similarity of the two images is obtained to determine the acceptability of the reconstructed method. When *K* = 1, the corresponding coordinates of the schlieren ball center in the main lobe image are first obtained via the four methods and then by image reconstruction. Finally, the correlation coefficient between the reconstructed images and the main lobe image are calculated, obtaining 0.629, 0.615 0.806 and 0.994. The correlation coefficient obtained using the self-correlation matching method is 0.994, and thus, the accuracy of the reconstructed image will be approximately 99.4%. The automatic reconstruction algorithm based on the self-correlation matching method is consistent and reliable, and the accuracy of the reconstructed image is verified. The mean values of the reconstructed image and of the main lobe image are 34.621 and 35.735, respectively, and the variances are 65.810 and 65.099, respectively, indicating the great similarity of the two images.

In previous targeting experiments, when seeking the best matching point for the schlieren ball center in the main lobe image, the general methods applied have been the calibration method [4], the center of gravity method [19] and the geometrical center method [13], all of which have obvious deficiencies. For the calibration method, because there is great vibration and atmospheric turbulence when conducting a shooting experiment, the experimental conditions of the light path between the shooting experiment and the calibration are completely different. If the splicing point obtained in the calibration laser path is replaced by the splicing points obtained in the shooting laser path, there will be large errors. For the center of gravity method and the geometrical center method, when the distribution of the main lobe is more asymmetric, there will be substantial error between the center of gravity and the best stitching point. The best matching point stitching algorithm, which is proposed in this paper, has the following advantages: 1) it can find the location of the stitching automatically, 2) the stitching error is greatly reduced, and 3) the mosaic trace of the stitching position is eliminated by the boundary fusion algorithm.

### Error analysis

Because there is background noise in the actual images captured by the CCDs, background noise must be considered during the process of automatic reconstruction. Under the assumption that the noise distribution functions of the main lobe and side lobe CCDs are *m*_*1*_*(x*_*1i*_, *y*_*1i*_*)* and *m*_*2*_*(x*_*2j*_, *y*_*2j*_*)*, respectively, the distribution function of the reconstructed focal spot is expressed as

$$\left(x_{i},y_{i}\right)=\{\begin{array}{ll} \frac{1}{k_{1}}\left[f_{1}\left(\frac{X_{1i}}{\alpha },\frac{Y_{1j}}{\alpha }\right)+m_{1}\left(\frac{X_{1i}}{\alpha },\frac{Y_{1j}}{\alpha }\right)\right] & \left(x,y\right)\in B\\ \frac{d_{1}}{k_{1}}\left[f_{1}\left(\frac{X_{1i}}{\alpha },\frac{Y_{1j}}{\alpha }\right)+m_{1}\left(\frac{X_{1i}}{\alpha },\frac{Y_{1j}}{\alpha }\right)\right]+\frac{d_{2}}{k_{2}}\left[f_{2}\left(\frac{X_{2i}}{\beta },\frac{Y_{2j}}{\beta }\right)+m_{2}\left(\frac{X_{2i}}{\beta },\frac{Y_{2j}}{\beta }\right)\right] & \left(x,y\right)\in B\cap C\\ \frac{1}{k_{2}}\left[f_{2}\left(\frac{X_{2i}}{\beta },\frac{Y_{2j}}{\beta }\right)+m_{2}\left(\frac{X_{2i}}{\beta },\frac{Y_{2j}}{\beta }\right)\right] & \left(x,y\right)\in C \end{array}$$ (5)

where *g* is the fused image; *f*_*1*_ and *f*_*2*_ are the main lobe and side lobe images, respectively; i and j are the coordinates of the CCD images; and *d*_*1*_ and *d*_*2*_ are the relative image proportions of the main lobe and side lobe in the overlapping region [5]. *d*_*1*_ varies gradually from 1 to 0 as *d*_*2*_ varies from 0 to 1: *d*_*1*_ + *d*_*2*_ = 1, 0< *d*_*1*_<1, and 0< *d*_*2*_<1.

In formula (5), we can see that the absolute value of the background noise is different in each region of the merged image. Even if the average value of the background noise is subtracted from the main lobe and side lobe images, the noise standard deviation in each region of the merged image is different.

$$\sigma \left(x,\text{y}\right)=\{\begin{array}{cc} \frac{\alpha^{2}\sigma_{m1}}{k_{1}} & \left(x,y\right)\in B\\ \frac{d_{1}\alpha^{2}\sigma_{m1}}{k_{1}}+\frac{d_{2}\beta^{2}\sigma_{m1}}{k_{2}} & \left(x,y\right)\in B\cap C\\ \frac{\beta^{2}\sigma_{m1}}{k_{2}} & \left(x,y\right)\in C \end{array}$$ (6)

where *σ*_*m1*_ and *σ*_*m2*_ are the noise standard deviations of the main lobe and side lobe CCDs and σ is the noise standard deviation of the reconstructed image. Because the gray level is obvious when the reconstructed image is merged directly, the fade in/fade out method is used to fuse the overlapping boundary [11,14]. The weighted value is calculated based on the distance between the current pixel coordinates and the boundary coordinates (the cross point of the splicing ring and the line, which connects the center of the splicing ring and the current pixel) [20].

### Analysis of the dynamic range

**Fig 10:**
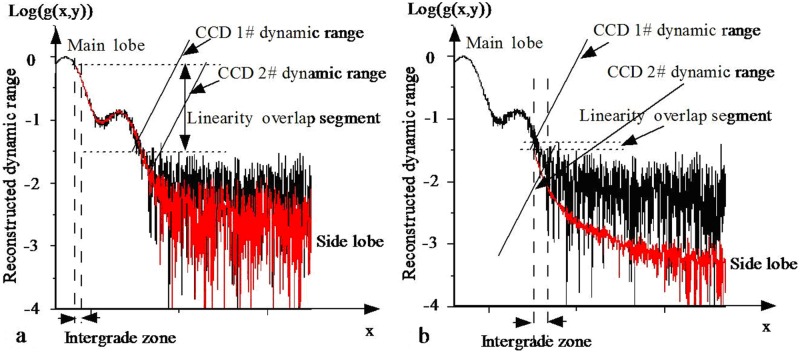
Dynamic range of the reconstructed focal spot. (a) *K*≈1. (b) *K*>>1.

The dynamic range [17,21] of the reconstructed image is relative to both the dynamic ranges of the two CCDs and the intensity attenuation ratio *K* (*K*>1) [22]. For the same peak value of the main lobe CCD, a smaller value of *K* results in a lower dynamic range and a smoother fused boundary (see Fig 10(a)), whereas a larger value of *K* results in a larger dynamic range and a more obvious splicing mark at the boundary. The rougher the merged image is in the splicing region, the larger is the error of the merged region (see Fig 10(b)). When *b* = 1 and *K* = 1, the linear region of the two CCD images is completely overlapped. The main lobe image is consistent with the side lobe image in the absence of the small ball. The distribution of background noise in the merged image is equivalent in each region, and the reconstructed image is smoothest in the overlapping region. When *K*>1, the linear overlapping region will decrease with increasing *K*, and the dynamic range of the reconstructed image will increase. However, the linear overlapping region will move to the linear lower limit of the main lobe CCD, and thus, the error of the fused boundary will increase, and the merged image will become rougher.

The dynamic range is defined as the ratio of the maximum gray value of the reconstructed image to the minimum valid gray value signal of the side lobe image. 90DL is the defined value of the minimum signal side lobe image, which is the gray mean value of a circle with a radius within 5 pixels of 1.5 times the small ball radius. The experimental data for the 3 groups are shown in Table 3.

**Table 3:** The dynamic range test results.

No.	Bits of scientific CCD	Experimental parameter	Maximum gray value of the reconstructed image	90DL	Dynamic range
1	14	b = 1.875 k = 150	492445	427.537	1151.8:1
2	14	b = 1.0 k = 132	530150	404	1312:1
3	16	b = 1 k = 11	686779	605.7	1133.8:1

As shown in the table, the measurement of a high-dynamic-range laser focal spot is implemented in 3 groups of experimental data. The measured dynamic range is greater than 1000:1, but there are huge differences in the light intensity attenuation ratio *K* because of different numbers of bits characterizing the CCD. The light intensity attenuation ratio obtained in experiments 1 and 2 is 10 times the intensity attenuation ratio obtained in experiment 3 because the measurement of the high-dynamic-range laser focal spot is mainly limited by the dynamic range of the scientific CCD. When the 14-bit scientific CCD is used to measure the far-field focal spot, the maximum gray value is 4095, and the dynamic range is only 100:1. To measure the far-field spot for which the maximum gray level is one hundred thousand or hundreds of thousands, the main lobe intensity will be attenuated by a factor of 25–250. Therefore, if the laser intensity of the splicing main lobe is magnified 25–250 times, a serious distortion of the reconstructed image will occur. The experimental results show that, when the intensity of the main lobe is magnified 1–10 times, the distortion is minimal, and the splicing effect is optimal. When the 16-bit scientific CCD is used in the actual experiment to measure the far-field spot, the maximum gray value is 65535. If the far-field focal spot for which the maximum gray level is hundreds of thousands is measured, the attenuation of the main lobe spot will only be 1–10 times. Thus, the distortion of the reconstructed image is very slight, and the splicing effect is optimized. However, 16-bit scientific CCDs are imported, and thus, they are more expensive. If the target energy is high and the attenuation is improper, a 16-bit scientific CCD will be damaged, which results in a high experimental cost.

Although the measurement of the far-field focal spot with a high dynamic range can be completed using the schlieren method, there are 3 shortcomings of this paper. 1) There is a certain deviation between the actual experimental results and the original expectations, and there is a severe distortion of the reconstructed focal spot, mainly because the intensity of the main lobe laser is magnified hundreds of times when the emission energy is excessive. As a result, it cannot accurately reflect the true spot distribution. In addition, the splicing step of the edges is obvious, and it is very difficult to obtain a satisfactory stitching effect. Moreover, because the small schlieren ball cannot block the side lobe spot center correctly during emission of the high-power facility, the edge of the side lobe spot is saturated. Thus, it is very difficult to identify the best matching point using the automatic reconstruction algorithm, and automatic matching will fail. 2) The first shortcoming described above is that the laser path is collimated by a simulated laser, namely, a beam emitted from a low-energy laser source when the total laser path is collimated and is different from the targeting laser, which is emitted from a high-power laser source of approximately 10000 J. The main lobe and side lobe spots are captured by the integrated diagnostic system. However, the actual experimental situation is such that the energy of the simulated laser is minimal. The simulated laser cannot reach the position of the side lobe CCD and main lobe CCD; thus, it is necessary to insert a relay light source at the position of the small target chamber to complete the collimation of the small target chamber behind the laser path, which increases the complexity and time requirement of the optical collimation. The main explanation for this is a lack of understanding of the design complexity of the optical path. 3) The mathematical model cannot consider the effect of noise on the reconstructed image. The noises of the two CCDs are different; thus, formula (8) in the focal spot distribution function is improved, and the impact of noise is analyzed in detail.

Based on the research presented in this paper, an integrated diagnostic system was designed to capture the main and side lobes of a far-field focused laser spot. The automatic reconstruction of the far-field focal spot was achieved. The least squares method was used to fit the center of the side lobe schlieren small ball. The findings will have very important significance in obtaining integrated and precise laser parameters.

## Conclusions

This paper presents an improved schlieren method for constructing high-dynamic-range images of far-field focal spots and improving the reconstruction accuracy and efficiency. The main lobe and side lobe beam spots are captured by two scientific-grade 14-bit CCD cameras at a 10 kJ-level laser facility, and the measurement of the far-field focal spot for a high-power laser facility is achieved using schlieren reconstruction. A self-correlation template matching algorithm is used to identify the optimal matching position and to reconstruct the focal spot by identifying the position with the largest correlation coefficient between the side lobe image and the main lobe image with a circle dug as the best matching position, thereby enabling the automatic splicing of the main lobe and side lobe images. In addition, the least squares method is used to fit the center of the side lobe schlieren small ball, and the error is less than 1 pixel. The experimental results show that the method can achieve an accurate, high-dynamic-range measurement of the far-field focal spot and automatic image reconstruction. The texture of the reconstructed image in the splicing region shows excellent agreement; not only is the stitching error reduced greatly, to less than one pixel, but the experimental efficiency for targeting experiments is also greatly improved.

## Supporting information

S1 File There are two important algorithms in the Supporting Information file: the first algorithm is “Calculating the center of the schlieren ball”, and the second algorithm is “Searching for the best matching point”, in which the code for the algorithms and a list of all variables used in the algorithm are provided. (DOC)
