# Supplementary material for: An improved schlieren method for measurement and automatic reconstruction of the far-field focal spot
Source: PLoS One. 2017 Feb 16;12(2):e0171415. doi: 10.1371/journal.pone.0171415 (PMC5312939; doi:10.1371/journal.pone.0171415)
Supplement: S1 File — (DOC) [file pone.0171415.s001.doc]

# 1 Calculating the center of the schlieren ball in the side lobe image

To calculate the center of the schlieren ball, the edge of the schlieren ball must first be obtained using the Sobel operator [1], and then, the least-squares method can be used to fit a circle with high fitting precision. According to the literature [2], the circle fitting formula is

$\begin{matrix} \left\{\begin{matrix} x_{0}=\frac{(\overline{x^{2}}\overline{x}+\overline{x}\overline{y^{2}}-\overline{x^{3}}-\overline{xy^{2}})((\overline{y})^{2}-\overline{y^{2}})-(\overline{x^{2}}\overline{y}+\overline{y}\overline{y^{2}}-\overline{x^{2}y}-\overline{y^{3}})(\overline{x}\overline{y}-\overline{xy})}{2((\overline{x})^{2}-\overline{x^{2}})((\overline{y})^{2}-\overline{y^{2}})-2(\overline{x}\overline{y}-\overline{xy})^{2}} \\ y_{0}=\frac{(\overline{x^{2}}\overline{y}+\overline{y}\overline{y^{2}}-\overline{x^{2}y}-\overline{y^{3}})((\overline{x})^{2}-\overline{x^{2}})-(\overline{x^{2}}\overline{x}+\overline{x}\overline{y^{2}}-\overline{x^{3}}-\overline{xy^{2}})(\overline{x}\overline{y}-\overline{xy})}{2((\overline{x})^{2}-\overline{x^{2}})((\overline{y})^{2}-\overline{y^{2}})-2(\overline{x}\overline{y}-\overline{xy})^{2}} \\ r=\sqrt{x_{0}^{2}-2\overline{x}x_{0}+y_{0}^{2}-2\overline{y}y_{0}+\overline{x^{2}}+\overline{y^{2}}} \end{matrix}\right. \\ \text{ } \end{matrix}$(1)

$\overline{x^{m}y^{n}}=\frac{1}{N}\sum_{i\in E} x_{i}^{m}y_{i}^{n}\text{ }$ (2)

where N is the total number of boundary points, $(x_{i},y_{i})$ represents the boundary coordinates of the side lobe image, and r is the radius. $(x_{0},y_{0})$ is the center of the schlieren ball; the center and radius of the schlieren ball in the side lobe image obtained from the circle fitting formula are (*Opx, Opy*) and *okr*, respectively.

| 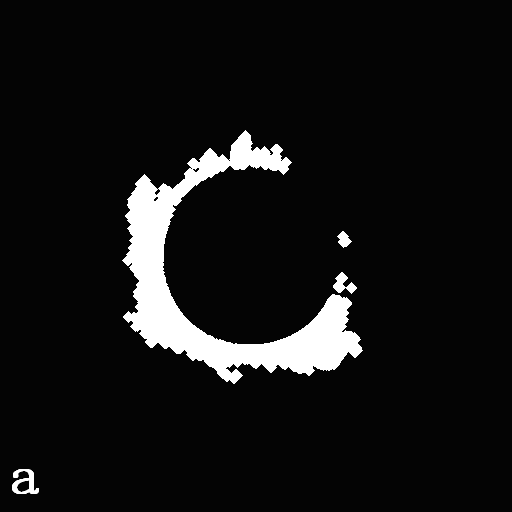 | 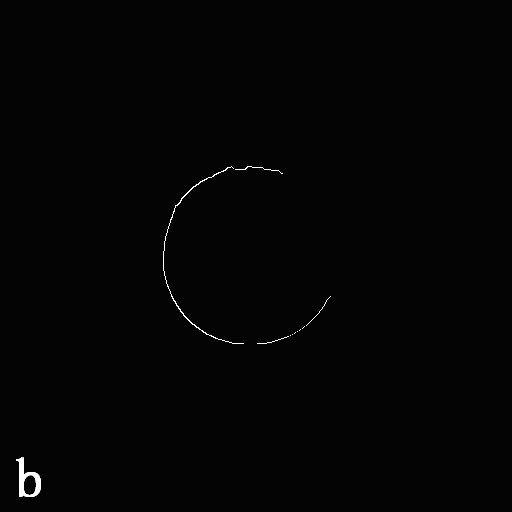 | 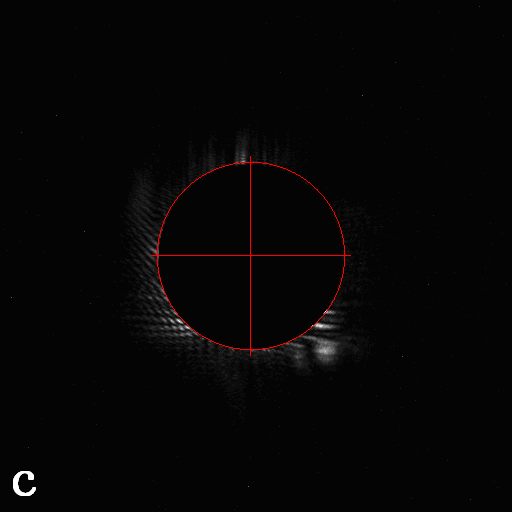 |
| --- | --- | --- |

**Fig 4.** Calculation of the center of the schlieren ball in the side lobe image. (a) The clipped image of the side lobe processed by means of morphological operation. (b) The edge of the small ball detected using the Sobel operator. (c) The final center and radius fitted using the circle fitting method

The input parameter of calculate the center of small ball is the edge image of small ball, which is detected using the Sobel operator. In obtain the center of small ball and radius, the code of circle fitting is written by C# as follows:

// a is the x coordinates of small ball center, b is the y coordinates of small ball center,

// and r is radius of small ball

int WID=512; //the width of image

Int HEI=512; // the width of image

int bytes = WID * HEI; // the size of image

byte[] grayValues = new byte[bytes];// save the edge image

int sum = 0;// save the count when gray value ==255

sum = 0;

for (int i = 0; i < HEI; i++)

{

for (int j = 0; j < WID; j++)

{

if (grayValues[i * WID + j] == 255)

{

sum = sum + 1;

}

}

}

// save all x and y coordinates of edge pixel in the array Ex and Ey array

double[] Ex = new double[sum];

double[] Ey = new double[sum];

int k = 0;

for (int i = 0; i < HEI; i++)

{

for (int j = 0; j < WID; j++)

{

if (grayValues[i * WID + j] == 255)

{

Ex[k] = j;

Ey[k] = i;

k++;

}

}

}

// calculate the x coordinates

//$x_{0}=\frac{(\overline{x^{2}}\overline{x}+\overline{x}\overline{y^{2}}-\overline{x^{3}}-\overline{xy^{2}})((\overline{y})^{2}-\overline{y^{2}})-(\overline{x^{2}}\overline{y}+\overline{y}\overline{y^{2}}-\overline{x^{2}y}-\overline{y^{3}})(\overline{x}\overline{y}-\overline{xy})}{2((\overline{x})^{2}-\overline{x^{2}})((\overline{y})^{2}-\overline{y^{2}})-2(\overline{x}\overline{y}-\overline{xy})^{2}}$

// a1—a18 is the calculating result of each item above formula.

POINTCOUNT = sum;

double a1, a2, a3, a4, a5, a6, a7, a8, a9, a10, a11, a12, a13, a14, a15, a16, a17, a18;

a1 = B(Ex, 1, Ex, 1) * Mean(Ex);

a2 = Mean(Ex) * B(Ey, 1, Ey, 1);

a3 = B(Ex, 1, Ex, 2);

a4 = B(Ex, 1, Ey, 2);

a5 = Mean2(Ey);

a6 = B(Ey, 1, Ey, 1);

a7 = B(Ex, 1, Ex, 1) * Mean(Ey);

a8 = Mean(Ey) * B(Ey, 1, Ey, 1);

a9 = B(Ex, 2, Ey, 1);

a10 = B(Ey, 1, Ey, 2);

a11 = Mean(Ex) * Mean(Ey);

a12 = B(Ex, 1, Ey, 1);

a13 = Mean2(Ex);

a14 = B(Ex, 1, Ex, 1);

a15 = Mean2(Ey);

a16 = B(Ey, 1, Ey, 1);

a17 = Mean(Ex) * Mean(Ey);

a18 = B(Ex, 1, Ey, 1);

double c1 = (a1 + a2 - a3 - a4) * (a5 - a6) - (a7 + a8 - a9 - a10) * (a11 - a12);

double c2 = 2 * (a13 - a14) * (a15 - a16) - 2 * (a17 - a18) * (a17 - a18);

double a = c1 / c2;

// calculate the y coordinates

//$y_{0}=\frac{(\overline{x^{2}}\overline{y}+\overline{y}\overline{y^{2}}-\overline{x^{2}y}-\overline{y^{3}})((\overline{x})^{2}-\overline{x^{2}})-(\overline{x^{2}}\overline{x}+\overline{x}\overline{y^{2}}-\overline{x^{3}}-\overline{xy^{2}})(\overline{x}\overline{y}-\overline{xy})}{2((\overline{x})^{2}-\overline{x^{2}})((\overline{y})^{2}-\overline{y^{2}})-2(\overline{x}\overline{y}-\overline{xy})^{2}}$

// b1—b18 is the calculating result of each item above formula.

double b1, b2, b3, b4, b5, b6, b7, b8, b9, b10, b11, b12, b13, b14, b15, b16, b17, b18;

b1 = B(Ex, 1, Ex, 1) * Mean(Ey);

b2 = Mean(Ey) * B(Ey, 1, Ey, 1);

b3 = B(Ex, 2, Ey, 1);

b4 = B(Ey, 1, Ey, 2);

b5 = Mean2(Ex);

b6 = B(Ex, 1, Ex, 1);

b7 = B(Ex, 1, Ex, 1) * Mean(Ex);

b8 = Mean(Ex) * B(Ey, 1, Ey, 1);

b9 = B(Ex, 1, Ex, 2);

b10 = B(Ex, 1, Ey, 2);

b11=a11;

b12=a12;

b13=a13;

b14=a14;

b15=a15;

b16=a16;

b17=a17;

b18 =a18;

double d1 = (b1 + b2 - b3 - b4) * (b5 - b6) - (b7 + b8 - b9 - b10) * (b11 - b12);

double d2 = 2 * (b13 - b14) * (b15 - b16) - 2 * (b17 - b18) * (b17 - b18);

double b = d1 / d2;

double r1, r2, r;

// calculate the radius r of small ball

//$r=\sqrt{x_{0}^{2}-2\overline{x}x_{0}+y_{0}^{2}-2\overline{y}y_{0}+\overline{x^{2}}+\overline{y^{2}}}$

r1 = a * a - 2 * Mean(Ex) * a + b * b - 2 * Mean(Ey) * b + B(Ex, 1, Ex, 1) + B(Ey, 1, Ey, 1);

r = Math.Sqrt(r1);

// calculate the result like $\overline{x^{2}}\overline{y}$

double B(double[] x,int m,double[] y,int n)

{

int M = POINTCOUNT;

double result=0;

for (int i=0;i<M;i++)

{

double a = Math.Pow(x[i], m) * Math.Pow(y[i], n);

result = result + a;

}

result = result / M;

return result;

}

// calculate the mean of array x

double Mean(double[] x)

{

int M = POINTCOUNT;

double result = 0;

for (int i = 0; i < M; i++)

{

result = result + x[i];

}

result = result / M;

return result;

}

// calculate the mean of array x

calculate the result like $\overline{x^{2}}$

double Mean2(double[] x)//

{

int M = POINTCOUNT;

double result = 0;

for (int i = 0; i < M; i++)

{

result = result + x[i] ;// Math.Pow(x[i], 2);

}

result = result / M;

result = result * result;

return result;

}

*Opx=x;*

*Opy=y;*

*Okr=r;*

# 2 The search for the best matching point of the schlieren ball center in the main lobe image

In the search for the best splicing position, a self-correlation template matching algorithm based on self-correlation matching theory is used [3]; the correlation coefficient of the two images is calculated as shown in formula (3):

(3)

where *f (x, y)* represents the image of size m × n, *w (x, y)* represents the sub-images of size *J× L*, and x=0,1,2,..., n-*L*, y=0,1,2,..., m-*J*. In the automatic reconstruction algorithm provided in this paper, *f (x, y)* represents the side lobe image, *w (x, y)* represents the main lobe image from which a circle with the same size as the schlieren ball is dug, and the size of the two images is 300 × 300.

The automatic reconstruction algorithm is described as follows:

| Algorithm 1 schlieren automatic reconstruction algorithm |
| --- |
| Input: *orgpb* image, *orgzb* image |
| 1 Get the center of gravity (*Ozx, Ozy*)of *orgzb,* the center(*Opx, Opy*) and radius *okr* of small schlieren ball in *orgpb.* |
| 2 Get the *cutpb* image, the center is (*Cpx,Cpy*), Get the *Pcir.* |
| 3 for m=-50: 50-1 |
| for l=-50: 50-1 |
| Get a *cutzb* image, *cutzb*=*orgzb*(*Ozy-150+m:Ozy+150-1+m,Ozx-150+l:Ozx+150-1+l*). |
| Dig a circle region from *cutzb* image,*cutzb*(i,j) is set to 0 when the *Pcir*(i,j) equal to 0. |
| end for |
| end for |
| 4 Get the best matching position *m,l* when the correlation coefficient between *cutzb* and *cutpb* is maximum. |
| 5 The the best matching *cutzb’* according to *m,l.* |
| 6 Fill the small ball region of *cutpb*,the data of *cutpb* located within the region of small ball is replaced by the data of *cutzb’* when the *Pcir*(i,j) equal to 0, and are amplified *K* times, the notation is *cutpb’*. |
| 7 The region of cutpb in orgpb is replaced by the final data of *cutpb’.* |
| 8 Fuse the splicing boundary by the weighted average method. |
| Ouput: The reconstructed image |

Where orgz*b* is the original main lobe image of size 512*512; org*pb* is the original side lobe image of size 512*512; *cutzb* is the cutting main lobe image of size 300*300; *cutpb* is the cutting side lobe image of size 300*300; *cutzb’* is the final cutting main lobe image;*cutpb’* is the final merged image with size of 300*300.

(*Ozx, Ozy*) is the center of gravity in *orgzb*;(*Opx, Opy*) and *okr* are the center and radius of schlieren ball in *orgpb*; (*Cpx,Cpy*) is the center of *cutpb.Pcir* is a matrix with 300*300, which mark the region of small ball of *cutpb.Pcir* (i,j) is set to 0, when (i,j) located within the region of small ball of *cutpb*, otherwise, *Pcir*(i,j) is set to 1.

The code of automatic reconstruction algorithm is written by Matlab 7.0, there are 7 files:

1) main_lobe.hig --the main lobe image

2) side_lobe.hig --the side lobe image

3) read_higfile.m --read .hig file

4) GetHigZbPbEx.m --read main_lobe.hig and side_lobe.hig together

5) imcorr.m --get the correlation coefficient between cutzb and cutpb

6) GetImgCenter.m --get the center of gravity of orgzb and orgpb image

7) Get_best_position_new.m --main function

The main function in file Get_best_position_new.m is shown as follows:

% The search for the best matching point of the schlieren ball center in the main lobe image

%Input: Opx- x coordinates of small ball center in orgpb image with size of 500*500

% Opy- y coordinates of small ball center in orgpb image with size of 500*500

% okr- radius of small ball

%Output: hb- The directly merged image with a size of 512×512

% hb1 the image fused the splicing region by weighted average method

% hb2 the splicing ring is shown in hb2 image.

close all;

Opx=251;

Opy=258;

okr=92;

[zb,pb]=GetHigZbPbEx(0); %the size of zb and pb is 1024*1024

[pbx,pby]=GetImgCenter(pb);

% get the center of gravity of side lobe original image

[zbx,zby]=GetImgCenter(zb);

zbx=uint16(zbx);

zby=uint16(zby);

pbx=uint16(pbx);

pby=uint16(pby);

% get the center of gravity of main lobe original image

zb=zb(zby-256:zby+256-1,zbx-256:zbx+256-1);

% get the orgzb image, the size is 512*512

yy=0;

xx=32;

pb=pb(pby-256:pby+256-1,pbx-256-xx:pbx+256-1-xx);

% get the orgpb image,and make the center of gravity is located in (255,255) around, the the size is 512*512,

max1=max(max(zb));

max2=max(max(pb));

figure(1);imshow(zb,[0 max1]);

figure(2);imshow(pb,[0 max2]);

%the background of side lobe image is subtracted from the side lobe iamge

bd=ones(512,512);

bd=bd*188; % substract the background from the pb, the mean gray value of background in pb image is 188

pb=pb-bd;

% the gray value of main lobe image is set to 0 when the gray value is less than 0

for i=1:512

for j=1:512

if pb(i,j)<0

pb(i,j)=0;

end

end

end

%the background of main lobe image is subtracted from the main lobe iamge

zbbd=ones(512,512);

zbbd=zbbd*184; % substract the background from the zb, the mean gray value of background in zb image is 184

zb=zb-zbbd;

% the gray value of main lobe image is set to 0 when the gray value is less than 0

for i=1:512

for j=1:512

if zb(i,j)<0

zb(i,j)=0;

end

end

end

orgzb=zb;

hold on

orgpb=pb;

hold on

% the center and radius of small schlieren ball are obtained by circle fitting method,

% the center is (cx,cy) , the radius is okr

cy=Opy;

cx=Opx;

plot(Opy,Opx,'*');

for i=1:512

for j=1:512

rr = (j - cx) * (j - cx) + (i - cy) * (i - cy);

if sqrt(rr)<okr

pb(i,j)=0;

end

end

end

maxpb=max(max(pb));

figure,imshow(pb,[0,maxpb]);title('The orgpb image of side lobe which a small schlieren ball is dug ')

[Ozx,Ozy]=GetImgCenter(zb);

pb_circle=zeros(300,300);

Ozx=uint16(Ozx);

Ozy=uint16(Ozy);

matflag=1;

if (matflag==1)

%%%%%%%%%%%%%%%%%%%%%%%%%%%%%%%%%%%%%

cutpb=pb(Opy-150:Opy+150-1,Opx-150:Opx+150-1);%the size of cutpb is 300*300

Cpx=151;

Cpy=151;

%okr=92;

maxcutpb=max(max(cutpb));

figure,imshow(cutpb,[0,maxcutpb]);;title('The cutpb image of size 300*300 ');

for i=1:300

for j=1:300

rr = (j - Cpx) * (j - Cpx) + (i - Cpy) * (i - Cpy);

if sqrt(rr)>okr

pb_circle(i,j)=1;

end

end

end

figure,imshow(pb_circle,[0,1]);;title('The cutpb circle of size 300*300 ');

cutzb=zb(Ozy-150:Ozy+150-1,Ozx-150:Ozx+150-1);%the size of cutzb is 300*300

maxcutzb=max(max(cutzb));

figure,imshow(cutzb,[0,maxcutpb]);title('The cutzb image of size 300*300');

tic

% search for the best matching point of the schlieren ball center in the

% main lobe image

WH=50;

matchxs=zeros(WH*2,WH*2);

for m=-WH:WH-1

for l=-WH:WH-1

% a circle region which the center is (cxzb,cxpb) and the radius is okr

% is dug from the cutzb image

cutzb=zb(Ozy-150+m:Ozy+150-1+m,Ozx-150+l:Ozx+150-1+l);

cutzb=cutzb.*pb_circle;

% calculate the correlation coefficients between cutpb image

% and all cutzb images

[Icorr,corry,corrx]=imcorr(cutpb,cutzb);

matchxs(WH+m+1,WH+l+1)=Icorr;

end

end

toc

% obtain the cutzb image which have the max orrelation coefficient between

% current cutzb image and cutpb image

max(matchxs(:))

[maxm,maxl]=find(matchxs==max(matchxs(:)));

m=maxm-WH-1;

l=maxl-WH-1;

% the best position is m=-16 and l=1;

%m=-16;

%l=1;

cutzb=zb(Ozy-150+m:Ozy+150-1+m,Ozx-150+l:Ozx+150-1+l);

cutzb1=cutzb;

cutzb=cutzb.*pb_circle;

figure,imshow(cutzb,[0,maxcutpb]);title('the cutzb image dug a small schileren ball when the best match positon is found');

[Icorr,corry,corrx]=imcorr(cutpb,cutzb);

fprintf('When m=%d l=%d, the correlation coefficient:%f\n is maximal.',m,l,Icorr);

%%%%%%%%%%%%%%%%%%%%%%%%%%%%%%%%

maxzb=max(max(cutzb));

figure,imshow(cutzb,[0,maxzb]);title('cutzb of main lobe image a small schileren is dug, (the best match position)')

for i=1:300

for j=1:300

if pb_circle(i,j)==0

cutpb(i,j)=cutzb1(i,j);

end

end

end

maxcutpb=max(max(cutpb));

figure,imshow(cutpb,[0,maxcutpb]);title('The directly merged image with a size of 300×300(1)');

% merge the main lobe and side lobe image into the reconstructed image

% directlly

hb=zeros(512,512);

hb=pb;

% hb(Opy-150:Opy+150-1,Opx-150:Opx+150-1)=cutpb(:,:);

for i=1:512

for j=1:512

rr = (j - cx) * (j - cx) + (i - cy) * (i - cy);

if sqrt(rr)<okr

hb(i,j)=zb(i+m,j+l);

else

hb(i,j)=pb(i,j);

end

end

end

maxhb=max(max(hb));

maxpb=max(max(pb));

figure,imshow(hb,[0,maxhb]);title('The directly merged image with a size of 512×512(2)');

% return;

% fuse the splicing region by weighted average method

hb1=zeros(512,512);

xs1=(0 :0.05:1);

xs2=1-xs1;

kd=10;

r=okr;

for i=1:512

for j=1:512

rr = (j - cx) * (j - cx) + (i - cy) * (i - cy);

if sqrt(rr)<okr

hb1(i,j)=zb(i+m,j+l);

else

hb1(i,j)=pb(i,j);

end

for k=1:10

if (sqrt(rr)>=r+k-1) &&(sqrt(rr)<=r+k )

a=pb(i,j);

b=zb(i+m,j+l);%*2

x1=xs1(k);

c=b*xs2(k)+a*xs1(k);

hb1(i,j)=c;%maxhb

end

end

end

end

maxhb1=max(max(hb1));

figure,imshow(hb1,[0,maxhb1]);title('The final constructed image fused the splicing region by weighted average method with size of 512*512');

% return ;

hb2=zeros(512,512);

%%%% the below code is to show the splicing ring

for i=1:512

for j=1:512

rr = (j - cx) * (j - cx) + (i - cy) * (i - cy);

if sqrt(rr)<okr

hb2(i,j)=zb(i+m,j+l);%*2

else

hb2(i,j)=pb(i,j);%+pbbd1

end

for k=1:1

if (sqrt(rr)>=r+k-1) &&(sqrt(rr)<=r+k )

a=pb(i,j);%+pbbd1

b=zb(i+m,j+l);%*2

x1=xs1(k);

c=b*xs2(k)+a*xs1(k);%

hb2(i,j)=maxhb;%maxhb

end

end

end

end

maxhb1=max(max(hb2));

figure,imshow(hb2,[0 maxhb1]);title('The position of splicing ring, the width is 2 pixels.');

%

hb1jbd_log =log10(hb1);

maxhb1jbd_log =max(max(hb1jbd_log))

figure,imshow(hb1jbd_log,[0 maxhb1jbd_log]);title('the result of merged image processed by the log function');

return ;

References

1. Mantiuk R, Myszkow SK, Seidel HP. A perceptual frame work for contrast processing of high dynamic range images. ACM Trans on Applied Perception. 2006;3(3): 288-308.

2. Kong B, Wang Z, Tan Y. Algorithm of laser spot detection based on circle fitting . The infrared and laser engineering.2001;31(3): 275-279.

3. Zhang Z, Wang YP, Xue GX. Digital image processing and machine vision——Visual C++ and Matlab implementation. Posts & Telecom Press, Beijing, 2013.
